# miR-125a attenuates the malignant biological behaviors of cervical squamous cell carcinoma cells through Rad51

**DOI:** 10.1080/21655979.2022.2051827

**Published:** 2022-03-25

**Authors:** Zeping Liu, Jinchang Huang, Qiuju Jiang, Xiaoling Li, Xiaohui Tang, Shasha Chen, Liling Jiang, Genghua Fu, Sijun Liu

**Affiliations:** a Department of Pathology, The Second Hospital of Longyan, Longyan, China; b Department of Pathology, Ganzhou People’s Hospital, the Affiliated Ganzhou Hospital of Nanchang University, Ganzhou, China; c Department of Gynaecology and Obstetrics, The Second Hospital of Longyan, Longyan, China; d Department of Pathology, The First Affiliated Hospital of Gannan Medical University, Gannan, China

**Keywords:** Cervical squamous carcinoma, microRNA-125a, Rad51, PI3K/Akt

## Abstract

Cervical squamous cell carcinoma (CSCC), the most common cervical malignancy, is more likely to invade and metastasize than other cervical cancers. miR-125a, a tumor suppressor gene, has been confirmed to be associated with cancer metastasis. However, the role of miR-125a in CSCC and the underlying mechanism are unknown. miR-125a expression was confirmed by real-time quantitative PCR (RT–qPCR), and the Rad51 expression level was measured by western blotting analysis. CSCC cell proliferation, migration and invasion were assessed with functional assays, including CCK-8, colony formation, wound healing and Transwell assays. Our data confirmed that miR-125a is expressed at low levels in CSCC tissues and cells. Functionally, the overexpression of miR-125a greatly prevented the proliferation, migration and invasion of CSCC cells, and the inhibition of miR-125a expression strongly enhanced these behaviors in CSCC cells. Moreover, the expression of Rad51, a miR-125a target gene, greatly reversed the miR-125-mediated inhibition of CSCC cell proliferation, migration and invasion. In addition, we discovered that miR-125a downregulated the levels of phosphorylated PI3K, AKT and mTOR through Rad51 in CSCC cells. miR-125a, a tumor suppressor, can attenuate the malignant behaviors of CSCC cells by targeting Rad51. Therefore, the miR-125a/Rad51 axis might be a target for CSCC therapy.

## Graphical abstract

**Figure:**
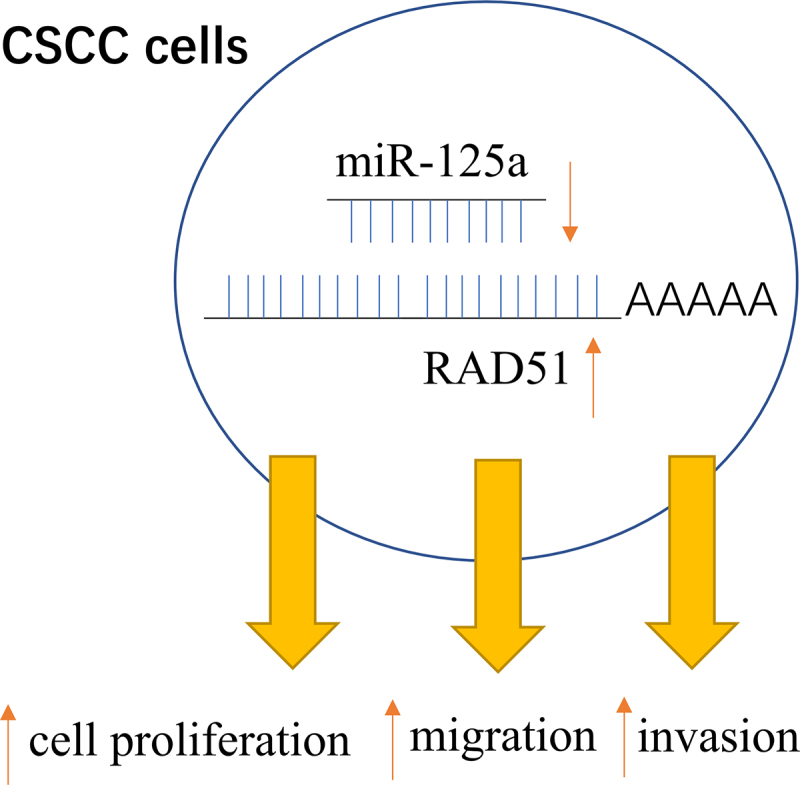

## Introduction

Cervical cancer is the most common malignancy of the female reproductive tract, and its worldwide annual incidence is second only to that of breast cancer [1]. The latest statistics indicate that approximately 538,000 new cases of cervical cancer and 266,000 cervical cancer-related deaths occur annually [2]. Based on the cell and tissue structure, cervical cancer is basically divided into invasive cervical squamous cell carcinoma (CSCC) and adenocarcinoma of the cervix (AC), and CSCC accounts for approximately 75–80% of cervical cancer cases [3]. Patients with early-stage (FIGO stage I–II) and locally infiltrated cervical cancer can achieve a five-year survival rate of greater than 80% with aggressive treatment [4]. In contrast, patients with advanced cervical cancer (FIGO stage III–IV) have a five-year survival rate of only 50–60%, and the five-year survival rate of patients with relapse or metastasis is less than 10% [5]. The vast majority of patients with advanced cervical cancer have CSCC [6]. To date, there is no effective treatment for CSCC patients, and recurrence and metastasis remain the primary reasons for the failure of CSCC therapy [7]. The recent development of targeted tumor therapy has brought new hope to patients with advanced cancer [8]. Therefore, the current trend in the clinical treatment of CSCC patients is to improve their prognosis and overall survival rate with treatments that function at the molecular level. The molecular mechanism underlying targeted therapy in CSCC is also the focus of this study.

MicroRNAs (miRNAs), small noncoding RNAs with a length of approximately 22–25 nucleotides, can bind to the 3’-UTRs of target mRNAs to negatively regulate their expression [9,10]. Different miRNAs can exert either oncogenic or tumor-suppressive effects, depending mainly on the regulation of downstream target genes or signaling pathways [11,12]. Recent studies verified that miRNAs are related to biological functions, such as proliferation, apoptosis, autophagy, hormone secretion, angiogenesis, and metastasis, in multiple cancers, including cervical cancer [^13–15^]. miR-125a, a tumor suppressor, has also been shown to be related to various cancers, including pancreatic cancer [16], breast cancer [17], gastric cancer [18], bladder cancer [19] and other cancers. However, the specific role of miR-125a in CSCC and the underlying mechanism have not been reported.

In this study, we further explored the change in the expression of miR-125a in CSCC tissues and cells and determined the effects of miR-125a on the proliferation, migration and invasion of CSCC cells. In addition, we further investigated the possible target genes and regulatory pathways of miR-125a in CSCC, aiming to further understand the roles of miR-125a in CSCC progression and to provide a feasible basis for CSCC diagnosis and therapy.

## Materials and methods

### Tissue samples

CSCC and adjacent nontumor tissues were collected from 20 CSCC patients who were diagnosed and treated at The Second Hospital of Longyan between 2018 and 2019. No participant had received surgery, radiotherapy or chemotherapy at another hospital prior to treatment. Patients with noncervical squamous cell carcinoma and patients with multiple primary malignancies were excluded. All the samples were stored in liquid nitrogen immediately after collection. Written informed consent was acquired. This study was approved according to the ethical standards of The Second Hospital of Longyan.

### Cell culture

Normal human cervical epithelial cells (HcerEpic; Cat. No. ATCC-1073) and SiHa cells (Cat. No. HTB-35) were obtained from ATCC. HCC-0214 cells were established according to previous research [20]. Cervical carcinoma specimens were incised aseptically and cultured in vitro with tissue culture methods, and a tumor cell growth curve was generated. The morphology of the cells was observed, and cell cycle analysis and chromosome analysis were performed. The expression of tumor markers (Keratine and PCNA) in the cell line was measured by immunocytochemical techniques. All the cells were grown in DMEM (Gibco) supplemented with 10% fetal bovine serum (FBS; Sigma) at 37°C in 5% CO_2_.

### Cell transfection

As described previously [21], the negative control (NC) mimic, miR-125a mimic, NC inhibitor, miR-125a inhibitor, Rad51 siRNA (si-Rad51) and Rad51 siRNA NC were purchased from GenePharma (Shanghai, China). Based on the experimental aim, SiHa and HCC-0214 cells were transfected with these oligonucleotides using Lipofectamine 3000 (Invitrogen) for 48 h.

### Real-time quantitative polymerase chain reaction (RT–qPCR) assay

Total RNA was extracted from CSCC tissues and cells with TRIzol reagent (Invitrogen, MA, USA). cDNA was synthesized with a reverse transcription kit (Takara, Tokyo, Japan) and amplified using SYBR Green qPCR Master Mix (DBI Bioscience, Germany). U6 was used as the internal reference for miR-125a. The relative expression level of miR-125a was determined by the 2^−ΔΔCT^ method [22]. All the primers were designed with Primer 5 and synthesized by Sangon Biotech (Shanghai, China).

### Western blotting analysis

Western blotting was used to measure protein expression [23]. Total protein was extracted from transfected CSCC cells or tissues with RIPA lysis buffer (Beyotime, China), and the protein concentrations were quantified with a BCA kit. Proteins (40 μg) in each group were separated by 10% SDS–PAGE and transferred to PVDF membranes (Millipore). After blocking with 5% skim milk, the membranes to which proteins had bound were incubated first with primary antibodies (1:1000) overnight at 4°C and then with secondary antibodies (1:2000, Abcam) for 1 h. The membranes were exposed and developed in a darkroom by the addition of ECL reagent (Pierce; Cat. No. 32,106). Anti-Rad51, anti-p-PI3K, anti-p-AKT and anti-p-mTOR antibodies were obtained from Abcam (USA).

### CCK-8 assay

SiHa and HCC-0214 cells were seeded in 96-well plates and transfected with oligonucleotides in accordance with the experimental aims. The cells in each group were continuously cultured at 37°C, and 10 μL of CCK-8 reagent (Dojindo, Japan) was added at 0, 24, and 48 h. After incubation for another 2 h at 37°C, the absorbance of each cell group was measured with a microplate reader [24].

### Colony formation assay

Transfected SiHa and HCC-0214 cells (500 cells/well) were evenly seeded in a 6-well plate and cultured under normal conditions for 14 days at 37°C. After washing with PBS, the CSCC cells were fixed with 4% paraformaldehyde for 20 min and stained with 0.2% crystal violet for 5 min. After washing with water, the 6-well plate was dried, scanned and photographed under a microscope [24].

### Wound healing assay

Cell migration was assessed using a wound healing assay, as previously described [25]. Transfected SiHa and HCC-0214 cells (3 × 10^5^ cells/well) were evenly spread in a 6-well plate and incubated at 37°C for 12 h. With 10-μL pipette tips, scratches were produced perpendicular to the back wall of the culture plate. After washing, the cells were further cultured in serum-free medium in a 37°C incubator for 48 h, and the results of the migration assay were then imaged.

### Transwell assay

Prepackaged Transwell chambers coated with 0.3 mg/mL Matrigel (BD Biosciences) were used. Transfected SiHa and HCC-0214 cells (5 × 10^5^ cells/well) were seeded in the upper compartment of the Transwell chamber, and medium supplemented with 20% FBS was added to the lower compartment of the Transwell chamber. Then, the cells that did not invade the lower compartment were removed by wiping with a sterile cotton swab. The invaded cells were fixed with 4% formaldehyde and stained with 0.2% crystal violet. The invaded cells were observed and counted under a microscope [24].

### Luciferase reporter assay

This assay was performed according to the previously reported method [26]. We constructed wild-type (WT) and mutant (Mut) Rad51 plasmids based on the predicted binding sites between miR-125a and the Rad51 3’-UTR using the psiCHECK-2 vector.

The primer sequences used to amplify WT-Rad51 were 5’-CCCTCGAGCATGGTGCCTTAGGAATGACTTGG-3’ (forward) and 5’-ATTTGCGGCCGCCTGTCACCCTGGCTGGAATGC-3’ (reverse); the primer sequences used to amplify Mut-Rad51 were 5’- CAAAGGGAATGGGTCTGTTGCACGCCTTTTTTTCTGTCAG −3’ (forward) and 5’- CTGACAGAAAAAAAGGCGTGCAACAGACCCATTCCCTTTG −3’ (reverse). Next, SiHa cells were cotransfected with the miR-125a mimic and either the WT-Rad51 or Mut-Rad51 plasmid with Lipofectamine 3000 (Invitrogen) for 48 h. The luciferase activity in each group was examined based on the instructions of the dual-luciferase assay kit (Promega).

### Statistical analysis

The data are presented as the mean ± SD values and were analyzed using SPSS 21.0 software (SPSS, Inc.). One-way analysis of variance was used for comparisons among multiple groups, and a paired or unpaired Student’s *t* test was used for comparisons between two groups. *P* < 0.05 was considered to indicate a statistically significant difference.

## Results

### miR-125a expression is significantly downregulated in CSCC tissues and cells

**Figure 1.**
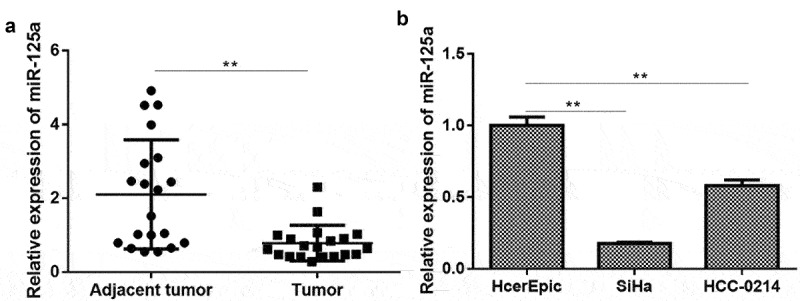
miR-125a expression is significantly downregulated in CSCC tissues and cells.

To further verify the role of miR-125a in CSCC and the underlying mechanism, we first measured the expression of miR-125a in CSCC. As shown in Figure 1a, the expression of miR-125a was significantly downregulated in CSCC tissues (n = 20) compared with adjacent nontumor tissues (n = 20) (*P* < 0.001). Similarly, we discovered that miR-125a expression in CSCC cells (SiHa and HCC-0214), especially in SiHa cells, was also significantly lower than that in normal human cervical epithelial cells (HcerEpic) (*P* < 0.01 (HCC-0214), *P* < 0.001 (SiHa); Figure 1b). In brief, we demonstrated that miR-125a is expressed at low levels in CSCC tissues and cells.

(a) The expression of miR-125a was assessed by RT–qPCR in CSCC tissues (n = 20) and adjacent nontumor tissues (n = 20). (b) The miR-125a level was also confirmed using RT–qPCR in normal human cervical epithelial cells (HcerEpic) and 2 CSCC cell lines (SiHa and HCC-0214). **P < 0.01, ***P < 0.001.

### miR-125a markedly suppresses the proliferation of SiHa and HCC-0214 cells

**Figure 2.**
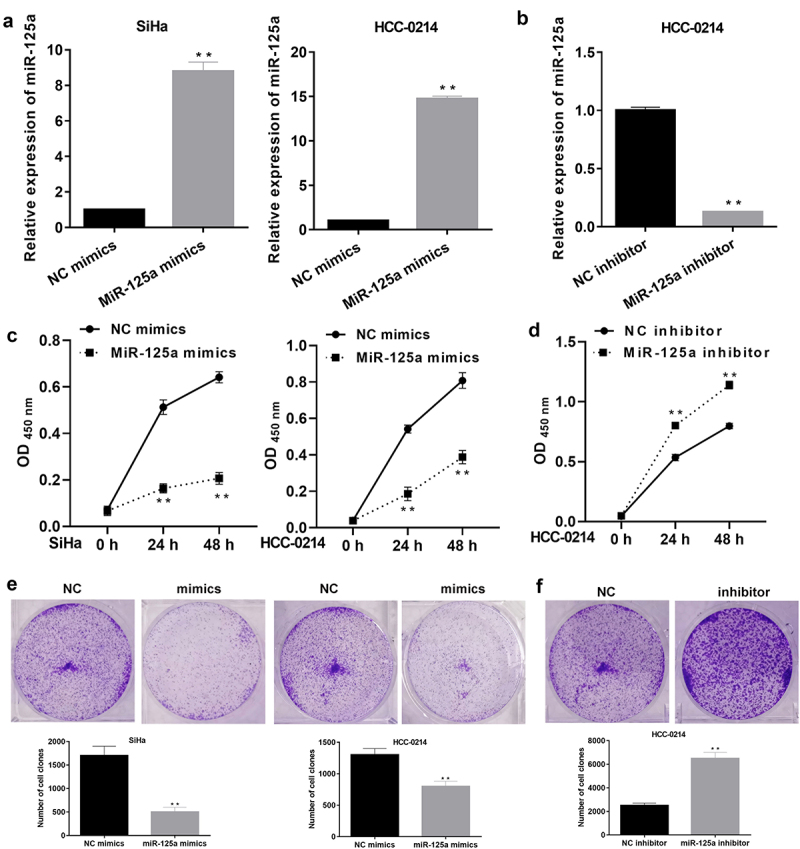
miR-125a markedly suppresses the proliferation of SiHa and HCC-0214 cells.

To further determine the influence of miR-125a on CSCC cell proliferation, miR-125a was overexpressed or inhibited in SiHa and/or HCC-0214 cells. Based on the above analysis of miR-125a expression in SiHa and HCC-0214 cells, miR-125a expression was dramatically downregulated in SiHa cells, indicating that the effect of silencing miR-125a expression in SiHa cells was not significant. Thus, we overexpressed miR-125a in SiHa/HCC-0214 cells and silenced miR-125a expression in HCC-0214 cells. The RT–qPCR data showed that in both SiHa and HCC-0214 cells, miR-125a expression was notably upregulated in the miR-125a mimic group relative to the NC mimic group (*P* < 0.001, Figure 2a), while in HCC-0214 cells, miR-125a expression was significantly downregulated in the miR-125a inhibitor group compared with the NC inhibitor group (*P* < 0.001, Figure 2b). Subsequently, the CCK-8 assay results showed that in SiHa and HCC-0214 cells, cell viability was markedly attenuated in the miR-125a mimic group compared to the NC mimic group (*P* < 0.001, Figure 2c), while in HCC-0214 cells, cell viability was notably enhanced in the miR-125a inhibitor group compared to the NC inhibitor group (*P* < 0.01, *P* < 0.001; Figure 2d). Similarly, the colony formation assay results also showed that overexpression of miR-125a markedly weakened the colony formation ability of SiHa and HCC-0214 cells (*P* < 0.01, *P* < 0.001; Figure 2e), while inhibition of miR-125a expression dramatically enhanced the colony formation ability of HCC-0214 cells (*P* < 0.01, *P* < 0.001; figure 2f). Overall, we proved that miR-125a can suppress CSCC cell proliferation.

(a) After SiHa and HCC-0214 cells were transfected with the miR-125a mimic or NC mimic, the level of miR-125a was verified by RT–qPCR. (B) The level of miR-125a was determined by RT–qPCR in miR-125a inhibitor-transfected HCC-0214 cells. (C) After miR-125a overexpression, SiHa and HCC-0214 cell viability was evaluated with a CCK-8 assay. (D) After miR-125a inhibition, HCC-0214 cell viability was assessed by a CCK-8 assay. (E) Cell colony formation was evaluated with a colony formation assay in miR-125a-overexpressing SiHa and HCC-0214 cells. (F) Cell colony formation was examined by a colony formation assay in HCC-0214 cells in which miR-125a was inhibited. **P < 0.01, ***P < 0.001.

### miR-125a greatly prevents the migration and invasion of SiHa and HCC-0214 cells

**Figure 3.**
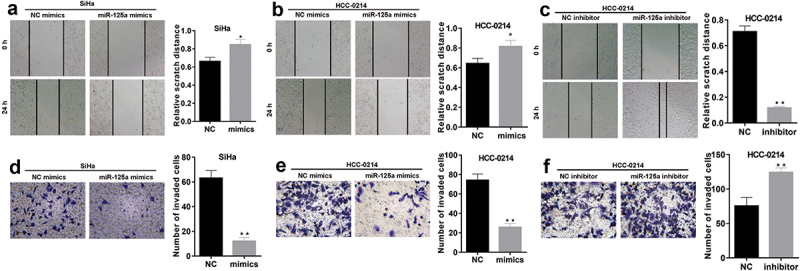
miR-125a dramatically suppresses the migration and invasion of SiHa and HCC-0214 cells.

We also verified the changes in the migration and invasion of CSCC cells after miR-125a overexpression or inhibition. The wound healing assay results showed that the relative wound width was significantly increased in miR-125a mimic-transfected SiHa and HCC-0214 cells compared to NC mimic-transfected cells (*P* < 0.05, Figure 3 a and b), but the relative wound width was markedly decreased in miR-125a inhibitor-transfected HCC-0214 cells compared to NC inhibitor-transfected cells (*P* < 0.001, Figure 3c). In addition, the Transwell assay results demonstrated that the numbers of invaded miR-125a mimic-transfected SiHa and HCC-0214 cells were notably lower than those of the corresponding NC mimic-transfected cells (*P* < 0.001, Figure 3 d and e), while the number of invaded miR-125a inhibitor-transfected HCC-0214 cells was notably higher than that of NC inhibitor-transfected HCC-0214 cells (*P* < 0.001, figure 3f). In summary, these data confirmed that miR-125a exerts significant inhibitory effects on CSCC cell migration and invasion.

The miR-125a mimic was transfected into SiHa and HCC-0214 cells, and the miR-125a inhibitor was transfected into HCC-0214 cells. (A-B) SiHa and HCC-0214 cell migration was investigated using a wound healing assay after miR-125a overexpression. (C) A wound healing assay was utilized to evaluate the migration of HCC-0214 cells after miR-125a inhibition. (D-E) The number of invaded miR-125a-overexpressing SiHa and HCC-0214 cells was assessed using a Transwell assay. (F) A Transwell assay was conducted to confirm the impact of miR-125a inhibition on the invasion of HCC-0214 cells. *P < 0.05, ***P < 0.001.

### Rad51 is a direct target gene of miR-125a

**Figure 4.**
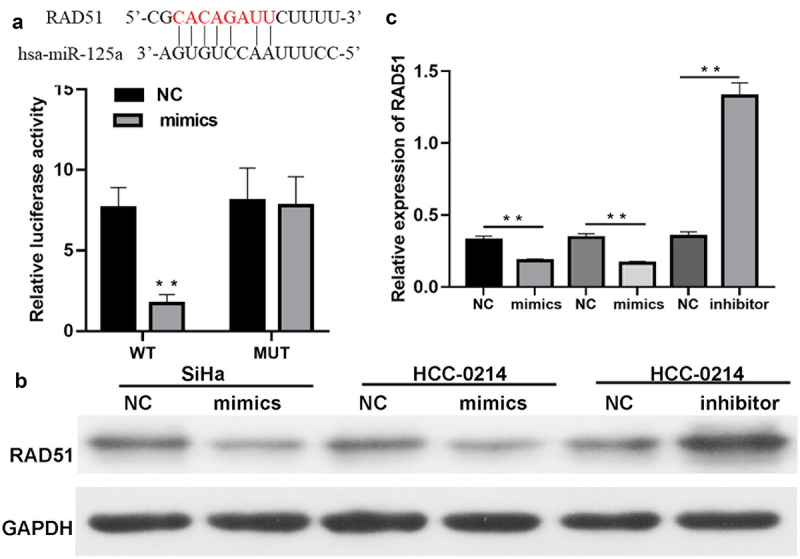
Rad51 is a direct target gene of miR-125a.

To explore whether miR-125a regulates Rad51 expression, we predicted the existence of binding sites between miR-125a and Rad51. The luciferase reporter assay results verified that miR-125a overexpression dramatically decreased the luciferase activity of WT-Rad51 but did not affect that of Mut-Rad51, proving the targeted regulatory effects of miR-125a on Rad51 (*P* < 0.01, Figure 4a). Next, we discovered that overexpression of miR-125a greatly downregulated Rad51 protein expression in SiHa and HCC-0214 cells and that inhibition of miR-125a expression markedly upregulated Rad51 protein expression in HCC-0214 cells (*P* < 0.001, Figure 4 b and c). Collectively, these findings showed that Rad51 is a target gene of miR-125a and that its expression is significantly downregulated by miR-125a in CSCC cells.

(a) We predicted and identified the binding sites between miR-125a and Rad51 and conducted a luciferase reporter assay to validate the direct targeting relationship of miR-125a and Rad51. (B) Western blotting analysis of Rad51 expression in SiHa and HCC-0214 cells in which miR-125a was overexpressed and HCC-0214 cells in which miR-125a was inhibited. (C) The relative expression level of Rad51 was calculated based on the gray values of the western blotting bands. **P < 0.01, ***P < 0.001.

### Rad51 is necessary for the suppressive effects of miR-125a on the proliferation, migration and invasion of SiHa and HCC-0214 cells

**Figure 5.**
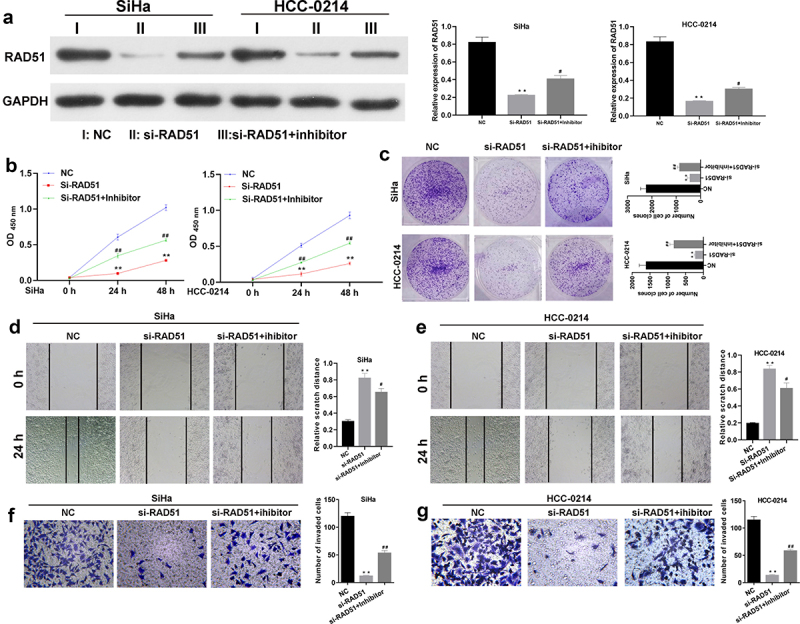
miR-125a markedly suppresses the proliferation, migration and invasion of SiHa and HCC-0214 cells by targeting Rad51.

To identify the impacts of miR-125a and Rad51 on the functions of SiHa and HCC-0214 cells. si-Rad51 and an miR-125a inhibitor were transfected into SiHa and HCC-0214 cells individually or in combination, and the change in the expression of Rad51 was investigated by western blotting analysis. As shown in Figure 5a, Rad51 expression was markedly reduced in the si-Rad51 group relative to the NC group but markedly elevated in the si-Rad51+ inhibitor group compared to the si-Rad51 group; these results suggested the successful transfection of si-Rad51 and the miR-125a inhibitor into SiHa and HCC-0214 cells. Then, the CCK-8 assay results revealed that Rad51 silencing significantly reduced the viability of SiHa and HCC-0214 cells, but the reduced cell viability due to Rad51 silencing was markedly reversed by the miR-125a inhibitor (*P* < 0.01, *P* < 0.001; Figure 5 a and b). Then, the colony formation assay results showed that Rad51 knockdown by siRNAs notably decreased the colony formation ability of SiHa and HCC-0214 cells, but this decreased colony formation ability was greatly reversed by the miR-125a inhibitor (Figure 5c). Additionally, the wound healing assay results indicated that knockdown of Rad51 expression notably increased the relative scratch distance in SiHa and HCC-0214 cells, while inhibition of miR-125a expression dramatically reversed this effect of Rad51 knockdown (*P* < 0.05, *P* < 0.001; Figure 5 d and e). Similarly, the Transwell assay results showed that knockdown of Rad51 expression markedly weakened the migration capacity of SiHa and HCC-0214 cells, while inhibition of miR-125a expression significantly reversed this effect of Rad51 knockdown (*P* < 0.001; figure 5 f and g). Collectively, our results demonstrated that the impacts of miR-125a on the proliferation, migration and invasion of CSCC cells are achieved through the regulation of Rad51.

SiHa and HCC-0214 cells were transfected with si-Rad51 and the miR-125a inhibitor individually or in combination. (A) Rad51 expression was confirmed by western blotting analysis. (B-C) Evaluation of transfected SiHa and HCC-0214 cell viability with a CCK-8 assay. (D) A colony formation assay was used to estimate the colony formation ability of each group. (E-F) The cell migration capacity was examined with a wound healing assay. (G-H) The cell invasion capacity was examined with a Transwell assay. ***P < 0.001 vs. the NC group; #*P* < 0.05, ##*P* < 0.01, ###*P* < 0.001 vs. the si-Rad51 group.

### Potential regulatory mechanism of miR-125a in CSCC cells by targeting Rad51

**Figure 6.**
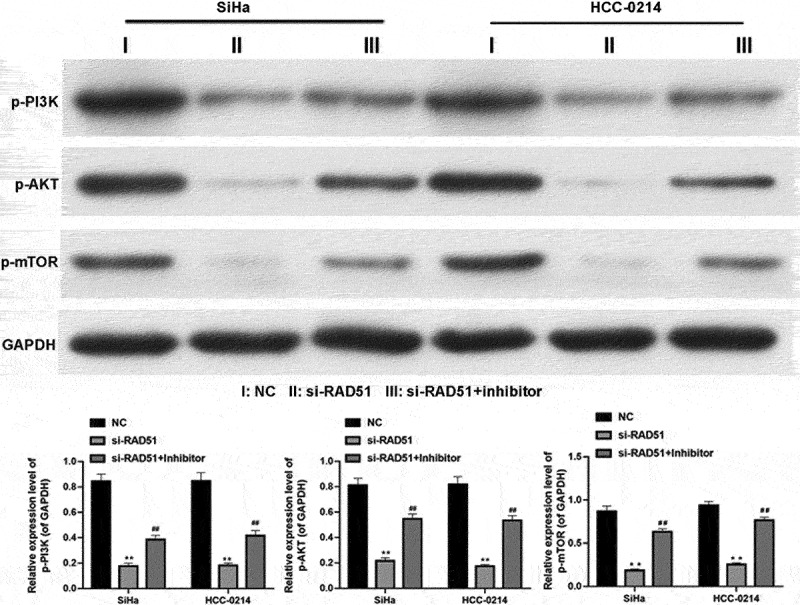
miR-125a clearly decreases PI3K and AKT phosphorylation and mTOR and IKK expression by targeting Rad51 in SiHa and HCC-0214 cells.

More importantly, we further explored the possible downstream regulatory proteins of the miR-125a/Rad51 axis in CSCC cells. The western blotting analysis results showed that the protein levels of p-PI3K, p-AKT and p-mTOR were dramatically reduced in Rad51-silenced SiHa and HCC-0214 cells compared with NC-transfected cells, while transfection of the miR-125a inhibitor markedly elevated the levels of these proteins that were reduced by Rad51 silencing in SiHa and HCC-0214 cells (*P* < 0.01, *P* < 0.001; Figure 6). Therefore, we verified that the PI3K/AKT/mTOR pathway can be regulated by the miR-125a/Rad51 axis in CSCC cells, indicating a possible mechanism by which the miR-125a/Rad51 axis functions in CSCC cells.

After SiHa and HCC-0214 cells were cotransfected with si-Rad51 and the miR-125a inhibitor, the levels of p-PI3K, p-AKT and p-mTOR were validated by western blotting analysis. The relative levels of these proteins were also quantified based on the gray values. ***P < 0.001 vs. the NC group; ###*P* < 0.001 vs. the si-Rad51 group.

## Discussion

Cervical cancer accounts for more than 50% of malignant tumors of female reproductive organs; CSCC accounts for approximately 75–80% of cervical cancer cases and is prone to invasion and metastasis [27]. Currently, most CSCC patients are hospitalized for treatment with surgery, radiotherapy, chemotherapy and other treatments; however, some CSCC patients experience recurrence and metastasis, which are seriously life-threatening [28]. Therefore, further investigation of the biological behavior and mechanism of CSCC and the identification of new molecular targets for CSCC therapy are of great practical significance.

miRNAs are a class of small single-stranded RNAs that are evolutionarily highly conserved [11]. Many studies have verified that dysregulation of miRNA expression is related to multiple cancer processes [10,29,30]. MiR-125 family members have been reported to function as tumor suppressor genes in many solid tumors, including gastric cancer [31], colorectal cancer [32], lung cancer [33], glioblastoma [34], etc. miR-125a is a member of the miR-125 family and is aberrantly expressed in multiple tumors [17,35]. In addition, downregulation of miR-125a expression has been correlated with tumor size, invasion, and lymph node metastasis in breast cancer [36]. Moreover, studies have suggested that miR-125a plays significant inhibitory roles in cancer cell metastasis in cancers such as non-small cell lung cancer [37], ovarian cancer [38], and colorectal cancer [39]. miR-125a has also been shown to be a marker for the suppression of proliferation and invasion in cervical cancer [40,41]. In our study, we further validated that miR-125a is expressed at low levels not only in CSCC tissues but also in CSCC cells. Additionally, we discovered that miR-125a can strongly suppress the proliferation, migration and invasion of CSCC cells. Thus, we suggest that miR-125a has an obvious suppressive effect on CSCC progression and might be a previously unidentified tumor suppressor gene in CSCC, and these findings might open new avenues for CSCC diagnosis and therapy.

miRNAs have been demonstrated to play their regulatory roles in cell biological processes by suppressing target genes [9]. A single miRNA can regulate multiple target genes, and a target gene can simultaneously be regulated by multiple miRNAs [30]. To investigate the target genes of miR-125a, we predicted the possible target genes with appropriate bioinformatics software tools. The prediction results indicated that Rad51 contains potential binding sites for miR-125a, indicating that it might be a target gene of miR-125a. The occurrence of most cancers is associated with DNA damage, and the efficacy of radiation and chemotherapy in tumor treatment depends mainly on causing DNA damage in tumor cells [42]. Homologous recombination (HR) repair is a vital DNA repair mechanism and plays a key role in maintaining the normal function and stability of genetic material [43]. The Rad51 protein is a key core enzyme responsible for DNA double-strand break (DSB) repair through HR [44]. Studies have proven that Rad51 is highly expressed in a range of solid tumors, such as colorectal cancer [45], breast cancer [46], prostate cancer [47], and pancreatic cancer [48]. Rad51 overexpression has been reported to cause the accumulation of DNA damage in precancerous cells; drive chromosome amplification, deletion and translocation; and lead to cancer development and metastasis [49]. In addition, Rad51 overexpression can also cause dysregulation of the cell cycle, resistance to apoptotic signals, resistance chemotherapeutic drugs, and radioresistance, thus promoting cancer progression [49]. Here, we demonstrated for the first time that miR-125a significantly downregulated Rad51 expression in CSCC cells. Moreover, our results confirmed that miR-125a can target the Rad51 3’-UTR, thereby downregulating Rad51 expression at the posttranscriptional level. We also found that miR-125a can suppress the proliferation, migration and invasion of CSCC cells by targeting Rad51.

Furthermore, our results proved that miR-125a can regulate the PI3K/AKT/mTOR pathway by targeting Rad51 in CSCC cells. The PI3K/Akt/mTOR pathway is the downstream signaling pathway of many signaling molecules, and its activation is associated with cell proliferation, invasion, metastasis, angiogenesis, apoptosis, etc [50,51]. Recently, numerous studies have verified that the PI3K/Akt pathway is active in cervical cancer and can cause the metastasis of cancer cells [52]. The PI3K/Akt pathway has also been confirmed to be related to CSCC progression [53,54]. Our results further indicated that the PI3K/AKT/mTOR pathway might be the underlying mechanism by which the miR-125a/Rad51 axis affects the functions of CSCC cells.

In summary, our current study revealed that miR-125a can suppress the proliferation, migration and invasion of CSCC cells by downregulating Rad51 expression to inhibit the PI3K/AKT/mTOR pathway. Therefore, the miR-125a/Rad51/PI3K/AKT/mTOR axis might be a therapeutic target in CSCC.

## Supplementary Material

Supplemental Material

## Data Availability

The datasets used and/or analyzed during the current study are available from the corresponding author on reasonable request.

## Abbreviations

CSCC, Cervical squamous cell carcinoma; RT–qPCR, real-time quantitative PCR; AC, adenocarcinoma of the cervix; miRNAs, MicroRNAs; WT, wild-type; HR, Homologous recombination
